# The Africa Ethics Working Group (AEWG): a model of collaboration for psychiatric genomic research in Africa

**DOI:** 10.12688/wellcomeopenres.16772.1

**Published:** 2021-07-27

**Authors:** Dorcas Kamuya, Mary A. Bitta, Adamu Addissie, Violet Naanyu, Andrea Palk, Erisa Mwaka, Eunice Kamaara, Getnet Tadele, Telahun Teka Wolde, Janet Nakigudde, Kiran Manku, Rosemary Musesengwa, Ilina Singh

**Affiliations:** 1 KEMRI-WELLCOME TRUST RESEARCH PROGRAMME, Kilifi, Kenya; 2 Clinical Research-Neurosciences, Centre for Geographic Medicine Research (Coast), KEMRI/Wellcome Trust Research Programme, Kilifi, Kenya; 3 Department of Psychiatry, University of Oxford, Oxford, UK; 4 Department of Preventive Medicine, School of Public Health, College of Health Sciences,, Addis Ababa University, Addis Ababa, Ethiopia; 5 Department of Sociology Psychology & Anthropology, School of Arts & Social Sciences, Eldoret, Kenya, Moi University, Eldoret, Kenya; 6 Department of Philosophy,, Stellenbosch University, Cape Town, South Africa; 7 Department of Anatomy, College of Health Sciences, Makerere University, Kampala, Uganda; 8 School of Arts and Social Sciences, Moi University, Eldoret, Kenya; 9 Department of Sociology, Addis Ababa University, Addis Ababa, Ethiopia; 10 Ministry of Science and Higher Education, Addis Ababa, Ethiopia; 11 College of Health Sciences, Makerere University, Kampala, Uganda

**Keywords:** Collaborative working groups; Research partnerships; Genomic research; Bioethics of psychiatric research

## Abstract

The Africa Ethics Working Group (AEWG) is a South-South-North collaboration of bioethics and mental health researchers from sub-Saharan Africa, working to tackle emerging ethical challenges in global mental health research. Initially formed to provide ethical guidance for a neuro-psychiatric genomics research project, AEWG has evolved to address cross cutting ethical issues in mental health research aimed at addressing equity in North-South collaborations. Global South refers to economically developing countries (sub-Saharan Africa in this context) and Global North to economically developed countries (primarily Europe, UK and North America). In this letter we discuss lessons that as a group we have learnt over the last three years; lessons that similar collaborations could draw on. With increasing expertise from Global South as an outcome of several capacity strengthening initiatives, it is expected that the nature of scientific collaborations will shift to a truly equitable partnership. The AEWG provides a model to rethink contributions that each partner could make in these collaborations.

## Disclaimer

The views expressed in this article are those of the author(s). Publication in Wellcome Open Research does not imply endorsement by Wellcome.

## Introduction

Research collaborations have proliferated within the global health research, as a response to the expanded mandate of global health to address the burden of disease in areas where expertise and capacity are still developing ( Berger *et al*., 2013; Ihekweazu *et al*., 2015; Mwangi *et al*., 2017; Noormahomed *et al*., 2017; O'Brien *et al*., 2018; Olapade-Olaopa *et al*., 2014) and in recognition that parachute research is exploitative ( Cash-Gibson *et al*., 2018; Kok *et al*., 2017) and out of touch with practical contextual issues. Mental health research, particularly that aimed at understanding pathophysiology, remains a global priority. However most of the research in this field is predominantly from the Global North ^1^, despite the burden of disease being disproportionately larger in the Global South ( GBD 2017 Risk Factor Collaborators, 2018). This leaves contribution from the Global South wanting.

Genomics is increasingly being used to understand the causes of mental illnesses, especially in the Global North ( Schizophrenia Working Group of the Psychiatric Genomics, 2014) and is slowly gaining traction in the Global South ( Gulsuner *et al*., 2020). North-South collaborations in psychiatric genomics research such as The Human Heredity and Health in Africa ( H3Africa, 2015), Neuropsychiatric Genetics of African Populations in Psychosis Study (NeuroGAP-P) ( Stevenson *et al*., 2019) and the Neuropsychiatric Genetics of African Populations in Neurodevelopmental Disorders Study (NeuroDev) ( de Menil *et al*., 2019) have been set up over the last decade. Such initiatives came into existence due to lack of representation from populations in the Global South and evidence about important interactions between genetics, psychiatric and neurodevelopmental illness, and the environment. These collaborations are funded by institutions in the Global North, and there is lack of clarity on who drives the research agenda. Further, the unique ethical challenges presented by psychiatric research, such as vulnerability of research participants due to stigma and structural inequalities, necessitates an exploration of ethical tensions in these collaborations.

In recognition of the need to create an independent ethics oversight group in the NeuroGAP-P ( Stevenson *et al*., 2019) and NeuroDev studies ( de Menil *et al*., 2019), the Africa Ethics Working Group (AEWG) was formed in 2016 ( Neurogene.org, 2017). In addition to providing the projects’ research teams with ethical guidance, the group explored and carried out empirical research on ethical dilemmas in psychiatric research in Africa with publications on vulnerability of participants ( Palk *et al*., 2020) and informed consent ( Kamaara *et al*., 2020). Current empirical work focuses on AEWG formation, beliefs towards saliva collection, biobanking, benefit sharing, ethics advisory structures, UBACC (University of California, San Diego, Brief Assessment of Capacity to Consent) and rapid ethical assessment. To our knowledge, the AEWG is the first ethics group created with the intention to drive procedural and substantive ethics research and advice within a major Global South-North psychiatric genomics collaboration. In this paper, based on experiences of the AEWG members, we outline four key lessons that could inform nature of collaborations in South-South and North-South collaborations, and the role of empirical ethics in informing critical ethical issues that collaborative research could consider.

### The Africa Ethics Working Group in Psychiatric Genomic Research (AEWG): a collaborative model

The AEWG was formed in February 2016, initially to support research teams of the NeuroGAP-P and the NeuroDev projects (henceforth referred to as genetics studies). The genetics studies are a collaboration between Broad Institute and Harvard T.H. Chan School of Public Health in the United States of America (USA), University of Oxford in the United Kingdom (UK), and various research institutes in Kenya, Uganda, Ethiopia and South Africa. The studies investigate potential genetic polymorphisms associated with psychotic and neuro-developmental disorders respectively. NeuroGAP-P aims to recruit a total of 35,000 participants, 17,500 cases and 17,500 controls, while NeuroDev will recruit 5600 participants, a third of these being cases.

Of the 15 members of AEWG, ten are based in the five sites undertaking psychiatric genetic studies; three part-time secretariat members and one co-opted member are based at the University of Oxford in the UK. The AEWG members come from diverse disciplines including experts in neuro-ethics, psychiatry, psychology, bioethics, philosophy, social science and public health. Most of the AEWG members are members of ethics review boards or committees either in their institutions (IRB) or/and at country level (ERC); 11 are based at universities while two are based at an internationally reputed research programme; 11 have PhDs and six hold either full or associate professorship posts in their institutions. Most of the AEWG members have an ethics background (undertaken as a course of study at master’s level and above); and have independent research grants, some of which are directly linked to the broad area of mental health (see https://neurogene.org/groups/aewg/).

### Paying attention to context in equitable collaborations: contribution of empirical ethics

Over the last decade, research collaborations in mental health between the Global North and South have proliferated both between (North-South) and within (South-South) groups leading to rapid growth in the understanding of health and illness across diverse settings ( Breuer *et al*., 2019; Lasalvia *et al*., 2015; Ramsay *et al*., 2016; Ramsay, 2015; Roberts *et al*., 2020; Thornicroft & Semrau, 2019; University of Cape Town, 2014). This has seen increased inclusion of the Global South in rapidly evolving fields such as psychiatric genomics and neuroscience with a promise to improve health outcomes. However, capacity for bioethics experts in the Global South remains low which has presented challenges in tackling ethical dilemmas that arise both from the field of mental health itself and from the collaborations ( Ramsay *et al*., 2014; de Vries *et al*., 2015). As a result, the Global South has remained vulnerable to inequity in mental health research partnerships and it is this gap that contributed to the formation of the AEWG.

It is common for ethics experts and committees to be viewed as auxiliary groups that offer ethical support to research projects but not as a discipline, deserving its own merit and respect and at par in disciplinary hierarchy with other scientific disciplines ( Sayers, 2007). This was not different at the initial formation of the AEWG, and raised questions about the autonomy of the AEWG and the degree to which it could independently influence research practices within the genomics research teams it was supporting. Indeed, some members voiced dilemma between supporting research investigators and fulfilling the ethics oversight role of the AEWG which complicated some critical functions of the group such as reporting protocol violations in meetings. Perhaps this situation contributed to AEWG evolving into an independent research group to allow it to empirically examine ethical questions that arose from the genetics studies but also to address topics such as equity in their collaboration. The result of this evolution has been the publication of a wide array of work that explores various issues that are applicable to many areas of mental health research such as the concept of vulnerability of psychiatric research patients and issues of translation and informed consent for psychiatric genomics research ( Kamaara *et al*., 2020; Kong *et al*., 2020; Palk *et al*., 2020).

### Paying attention to various forms of contribution in equitable collaborations

The evolution of the AEWG has not been without the challenges that underpin formation of new partnerships particularly those that involve unequal partners in terms of funding sources and the capacity of experts ( Munung *et al*., 2017; The Lancet Global Health, 2018). Although the AEWG’s main membership exclusively comprises members from sub-Saharan Africa, experts in ethics, the Secretariat that coordinates the overall functioning of the group is based in the Global North. This raises the question about equity in partnerships, and how practical it is, given skewed funding. It also raises questions about the northern collaborators’ view of their southern counterparts in terms of the latter’s level of expertise. For instance, members of the AEWG described the tensions that arose in determining the research agenda for AEWG. Whereas the partners from the Global North saw ethical dilemmas as opportunities for research, partners from the Global South felt that presenting their sites’ ethical dilemmas as research questions would be at conflict with their supportive role and would paint their sites in bad light. However, by viewing themselves as a research team rather than as a support team to the genetics research studies, the AEWG has been able to confront these questions using empirical methods as shown in their published and on-going work.

### Rethinking effectiveness in collaborative projects: not just metrices but also nature of collaboration

Unlike research teams which measure the success of their projects against the set objectives, important factors that make the collaboration work effectively can be overlooked, including for example coordination, managing relationships and building a feeling of collegiate. All these can lead to a shared understanding of the common goals, and to co-creation of knowledge while also providing opportunities to learn from each other. For the AEWG, in addition to paying attention to these factors, conducting primary research has been prolific, publishing in a wide array of topics within the field of mental health. Thus, the success of the group could be attributed to a combination of factors: (i) the genetics studies took off successfully and were able to continue which reduced the frequency of consultations with the ethics teams, (ii) the group drew on the wide range of skills and expertise within the members and the site principal investigators (PIs) to assist each other and in anticipating ethical issues and planning mitigation strategies, (iii) reimbursement for time and provision of small research support funds helped members empirically investigate ethical issues in context, (iv) frequent and scheduled correspondence including face to face meetings may have given members a sense of accountability and collegiate, (v) support from the investigators of the genetics studies who were involved in recruiting some members of the group, (vi) effective leadership and support from the Oxford based group. However, what remains unknown is the sustainability of this trend beyond the life of the genetics studies, that is, when funding is withdrawn. We believe that AEWG’s unique focus in global mental health and their track record as an independent research group places them strategically as competitive applicants for bioethics research funds and as co-applicants in other global mental health calls and it may sustain.

## Conclusion

Great attention to collaborations in global health research emphasise importance of equity, particularly in North-South collaborations. Less discussed is the nature of South-South collaborations and disciplinary contributions in such collaborations. Drawing on our experiences as ethics experts in the Global South working with Northern partners and embedded within psychiatric genomic research, this papers highlights some of lessons we have learnt over the last three years, which can help strengthen research collaborations. With increasing expertise from Global South and funding from their governments, it is expected that the nature of scientific collaborations will shift to a truly equitable partnership. The AEWG provides a model to rethink contributions that each partner could make in these collaborations, providing a knowledge exchange that is bi-directional.

## Data availability

No data are associated with this article.

## Footnotes

^1^ Global South refers to economically developing countries (sub-Saharan Africa in this context) and Global North to economically developed countries (primarily Europe, UK and North America)
