# Supplementary material for: The Africa Ethics Working Group (AEWG): a model of collaboration for psychiatric genomic research in Africa
Source: Wellcome Open Res. 2021 Jul 27;6:190. [Version 1] doi: 10.12688/wellcomeopenres.16772.1 (PMC8753570; doi:10.12688/wellcomeopenres.16772.1)
Supplement: Peer review history

# Peer review: Reviewer response for version 1

**DOI:** 10.21956/wellcomeopenres.18497.r47552

**Published:** 2022-01-11

**Authors:** Ruth Chadwick

**Affiliations:** 1 School of Social Sciences, Cardiff University, Cardiff, UK

This Open Letter explains very clearly both the need for and the challenges facing the Africa Ethics Working Group. It is more than adequately supported by relevant literature. One suggestion for improvement is that in the Introduction it is said that there are four key lessons. A more explicit reference back to the four key lessons in the Conclusion would perhaps have strengthened this message. There are some very minor issues - for example, on page 4, the word 'collegiate' does not sound quite right and could be replaced by 'collegiality'.

 Another point is that the level of discussion remains at a very abstract level, which is arguably inevitable in a piece of this length, but in a longer piece, a box with a possibly fictional case study illustrating good practice would be helpful, particularly regarding how things should develop in future.

Does the article adequately reference differing views and opinions?

Yes

Are all factual statements correct, and are statements and arguments made adequately supported by citations?

Yes

Is the Open Letter written in accessible language?

Yes

Where applicable, are recommendations and next steps explained clearly for others to follow?

Yes

Is the rationale for the Open Letter provided in sufficient detail?

Yes

Reviewer Expertise:

Ethics, Gene-ethics

I confirm that I have read this submission and believe that I have an appropriate level of expertise to confirm that it is of an acceptable scientific standard.

# Peer review: Reviewer response for version 1

**DOI:** 10.21956/wellcomeopenres.18497.r47555

**Published:** 2022-01-07

**Authors:** Alice Virani

**Affiliations:** 1 Department of Medical Genetics, The University of British Columbia, Vancouver, BC, Canada

This letter nicely outlines the context, challenges and need for AEWG. The paper also adequately summarizes the formation, evolution and work of the AEWG. The paper and group could be strengthened by further comment on how they involve and include voices of stakeholders such as patient groups, advocacy groups and communities. Finally, further discussion on how the group will move forward/future projects would be helpful.

Does the article adequately reference differing views and opinions?

Yes

Are all factual statements correct, and are statements and arguments made adequately supported by citations?

Yes

Is the Open Letter written in accessible language?

Yes

Where applicable, are recommendations and next steps explained clearly for others to follow?

Yes

Is the rationale for the Open Letter provided in sufficient detail?

Yes

Reviewer Expertise:

Ethics, genomics, research ethics

I confirm that I have read this submission and believe that I have an appropriate level of expertise to confirm that it is of an acceptable scientific standard.
